# Involvement of Alarmins in the Pathogenesis and Progression of Multiple Myeloma

**DOI:** 10.3390/ijms22169039

**Published:** 2021-08-21

**Authors:** Giuseppe Murdaca, Alessandro Allegra, Francesca Paladin, Fabrizio Calapai, Caterina Musolino, Sebastiano Gangemi

**Affiliations:** 1 Department of Internal Medicine, University of Genoa, Ospedale Policlinico San Martino IRCCS, 20132 Genoa, Italy; puell-a@hotmail.it; 2 Division of Hematology, Department of Human Pathology in Adulthood and Childhood “Gaetano Barresi”, University of Messina, 98125 Messina, Italy; aallegra@unime.it (A.A.); cmusolino@unime.it (C.M.); 3 Department of Chemical, Biological, Pharmaceutical and Environmental Sciences, University of Messina, 98168 Messina, Italy; fabrizio.calapai@unime.it; 4 Department of Clinical and Experimental Medicine, School and Operative Unit of Allergy and Clinical Immunology, University of Messina, 98125 Messina, Italy; gangemis@unime.it

**Keywords:** alarmins, cytokines, multiple myeloma

## Abstract

Objective: Multiple Myeloma (MM) is a haematological disease resulting from the neoplastic transformation of plasma cells. The uncontrolled growth of plasma cells in the bone marrow and the delivery of several cytokines causes bone erosion that often does not regress, even in the event of disease remission. MM is characterised by a multi-step evolutionary path, which starts with an early asymptomatic stage defined as monoclonal gammopathy of undetermined significance (MGUS) evolving to overt disease. Data Sources and Study Selection: We have selected scientific publications on the specific topics “alarmis, MGUS, and MM”, drawing from PubMed. The keywords we used were alarmines, MGUS, MM, and immune system. Results: The analysis confirms the pivotal role of molecules such as high-mobility group box-1, heat shock proteins, and S100 proteins in the induction of neoangiogenesis, which represents a milestone in the negative evolution of MM as well as other haematological and non-haematological tumours. Conclusions: Modulation of the host immune system and the inhibition of neoangiogenesis may represent the therapeutic target for the treatment of MM that is capable of promoting better survival and reducing the risk of RRMM.

## 1. Introduction

Multiple Myeloma (MM) is a haematological disease resulting from the neoplastic transformation of plasma cells, which are the terminally differentiated cells of the B lymphocyte line [1,2]. Malignant plasma cells can invade not only primarily the bone marrow but also peripheral blood and viscera in advanced widespread [3]. Plasma cells physiologically produce immunoglobulin (Ig). Malignant plasma cells produce and release excess monoclonal protein (M protein) in serum, which is also known as paraprotein [4]. The M protein will be excreted in the urine. Numerous genetic alterations favor the onset of the uncontrolled proliferation of plasma cells and the production of paraprotenin [1,2]. The uncontrolled growth of plasma cells in the bone marrow and the production of cytokines cause bone erosion, and the resulting bone lesions often do not regress, even in the event of disease remission. MM is the second most common haematological malignancy, and it is characterised by the appearance of bone pain, hypercalcemia, anemia, and renal failure [1,2,3]. MM is characterised by a multi-step evolutionary path, which starts with an early asymptomatic stage defined as monoclonal gammopathy of undetermined significance (MGUS) evolving to overt disease. MGUS is classified according to the secretion of Ig and, thus, into MGUS IgM and MGUS non-IgM. Although MGUS in most cases has a benign course, in some cases, it can evolve into aggressive forms. In particular, IgM MGUS can develop in Waldenström macroglobulinemia (WM) or, in fewer cases, in other non-Hodgkin’s lymphomas, while non-IgM MGUS originating from mature plasma cells can develop in MM [5]. MGUS can also secrete only the κ or λ light chain of Ig. Light chains aggregating are the cause of organ damage (i.e., heart, kidneys) both by depositing themselves or by favoring the deposition of amyloid from light chains (AL) [6,7]. However, in patients with MGUS, the M protein is <30 g/L and represents 20%–70% of all Ig, and circulating monoclonal plasma cells are <3 × 10^6^ per liter [8]. The advent of new drugs such as proteasome inhibitors and immunomodulatory agents have certainly improved the prognosis and median overall survival of MM to over 60 months [9]. However, MM still remains an incurable disease. The clinical picture and its progression over time is favored and aggravated by the inevitable onset of drug resistance [10,11,12,13]. Plasma cell dysfunction and uncontrolled proliferation, granulocytopenia both from tumour marrow invasion and iatrogenic from chemotherapy, and high-dose administration of dexamethasone promote immunodeficiency in the patient with MM. Immunodeficiency favors both recurrent opportunistic infections and the evasion of the tumour from the immune response with its progression and wide spread [10,14,15,16,17]. Immunodeficiency is the consequence of both the weakening of B- and T-lymphocyte response, but also the antigen-presenting cells (APC); the natural killer (NK) cells are compromised in their number and functionality [10,18,19,20,21,22,23,24].

The previously reported alterations of the effectors of the immune system at the bone marrow level seem fundamental not only in the determinism of the disease but also in its progression. In MM, the crucial function of the BM tumour microenvironment and in particular in the effectors of the immune system contained therein is well recognised, and numerous reports have reported that plasma cells intensely depend on it [25]. MM plasma cells cooperate, directly and indirectly, with the bone marrow milieu stimulating growth, survival, and chemoresistance [26].

Moreover, a recent investigation revealed that in the BM, MM mesenchymal stem cells (MSCs) have a specific gene expression profile with respect to the normal MSCs. The evaluation of the gene co-expression network demonstrated that the principal altered activities in MM-MSCs are correlated to cell cycle progression and modifications of the immune response, which may participate in MM physiopathology [27]. 

Furthermore, several findings demonstrated that tumour progression in MM is linked to a change of tumour-specific immunity [28], proposing that immune surveillance may have an action in the prevention of MM disease evolution, as communications with the contiguous milieu are essential to MM cells’ survival [29]. BM immune effector interactions with plasma cells are essential for MM evolution, as contacts increase the generation of different anti-apoptotic and cell cycle-stimulating factors [30]. Plasma cells use numerous mechanisms to escape immune surveillance, which comprise modified interactions with T-cells, dendritic cells, and natural killer cells. These changes can be due to immunosuppressive cells and cytokines such as IL-10, TGFβ, and IL-6 as well as a reduction of the antigen processing machinery [31]. 

Researchers’ attention focused on potential risk factors for the evolution of MGUS into MM. What factors might vary the quantity and type of protein M and the ratio of free immunoglobulin chains and induce the suppression of the production of polyclonal immunoglobulins (PIg), which is considered an undoubted factor of progression? What is the real role of alarmins in disease progression? The increased risk of contracting opportunistic infections has a complex genesis in which the dysregulation of cytokines undoubtedly plays an important role also because it promotes immune paresis [32,33]. The role that alarmins and cytokines play in the crosstalk between cells of the medullary stroma is now accepted, and thus, we have decided to analyse their role in the progression of MM. We hypothesise that alarmins play a role in the progression from MGUS to MM. We describe the role of alarmins and their potential in predicting whether a patient is at risk of rapid progression from MGUS to MM and finally whether they can be the target for future targeted therapies.

## 2. Alarmins

Alarmins are endogenous proteins or peptides which are substitutively expressed and play a chemotactic role. Alarmins are released after degranulation, cell damage, or death, and they activate the immune system. Alarmins interact with chemotactic and pattern recognition (PRR) receptors [34]. The excess release of alarmins represents a critical situation comparable to a cytokine storm with potentially lethal outcomes. It is well established that the expression “damage-associated molecular models (DAMP)” can be used interchangeably with that of “alarmin” [35]. The well-established hypothesis is that the immune system responds to DAMPs following an infection, resulting in cell damage and death [36]. Alarmins can be considered as a subset of endogenous DAMPs which similarly to cytokines bind to PRRs such as Toll-like receptors (TLRs) and play a role in host defense. The term of alarmins also includes the endogenous fractions generated with unscheduled cell death that induce innate/inflammatory and adaptive immune responses [37,38,39,40,41,42]. Among alarmins, defensins (α, β), cathelicidin/LL-37, high-mobility group box-1 (HMGB1), and heat shock proteins (HSPs) IL-1α, IL-33, and S100 proteins stimulate neutrophil granulocytes and monocytes/macrophages to release inflammatory mediators including tumour necrosis factor (TNF)-α, interleukin (IL)-1β, IL-6, IL-8, CCL2, CCL3, and CCL4, leukotriene B4, and nitride oxide [43,44,45,46].

### 2.1. High-Mobility Group Box-1 (HMGB1) 

HMGB1 belongs to HMG, which is a group of proteins with low molecular weight and high gel mobility, which is able to bind the non-histone chromosome component in eukaryotic cells [47,48]. The HMGB1 gene is located on chromosome 13q12 and includes five exons and four introns. HMGB1 stabilises the nucleosome, and it plays a pivotal role in DNA arrangement, replication, damage repair, and transcription by binding to DNA [49]. Necrotic tissue or cells under stress releases HMGB1, which by binding to PRR consequently induces DAMP [50]. Infections and injury represent the initial stimulus for the active release of HMGB1 from cells by activated immunity cells or passive from damaged or necrotic cells [51,52]. HMGB1 recalls neutrophils, induces the secretion of proinflammatory cytokines such as TNF-α and IL-6, activates dendritic cells (DCs) and released from necrotic cells acts as a marker of cell death [53,54]. HMGB1 acts by binding to certain receptors including RAGE, TLR (TLR2, TLR4, and TLR9), CXCR4, and mucin-3 T-cell immunoglobulin (TIM-3) [55,56].

### 2.2. Heat Shock Proteins (HSPs)

HSPs include several subfamilies (i.e., HSP27, HSP70, HSP90) which are involved in protein folding and protein regulation of homeostasis, preventing the formation of non-functional protein structures [57,58]. Some of the M proteins secreted by MM cells open or fold incorrectly and are lethal to MM cells. HSPs, by converting these proteins back into a ‘folding-competent’ state, allow the survival of these MM cells [59]. IL-1 (IL-1α and IL-1β) has a role both in inflammatory processes and in the immune response to infectious agents by binding to its receptor. Dysregulated production and signalling of IL-1 worsens tissue damage in infections and chronic diseases, including cancers [60]. IL-33, a member of the IL-1 family constitutively expressed by endothelial and epithelial cells, seems to guide the T helper cell 2 (Th2) response in several organs [61,62,63,64].

### 2.3. S100 Proteins

S100A8 and S100A9 are calcium-binding proteins mainly secreted by granulocytes and monocytes. The heterodimer (S100A8/S100A9) also known as calprotectin has the greatest proinflammatory potential. S100A9 is involved in many biological processes including inflammation, migration, invasion, and angiogenesis [65]. S100A9 promotes the production of reactive oxygen species (ROS), and it interacts with RAGE and TLR4, triggering the inflammatory cytokine cascade including TNF-α, IL-1β, IL-6, and IL-8 and, finally, it promotes tumour metastatic spread and immunosuppression by invoking the myeloid-derived suppressor cell (MDSC) [66,67,68]. 

**Figure 1:**
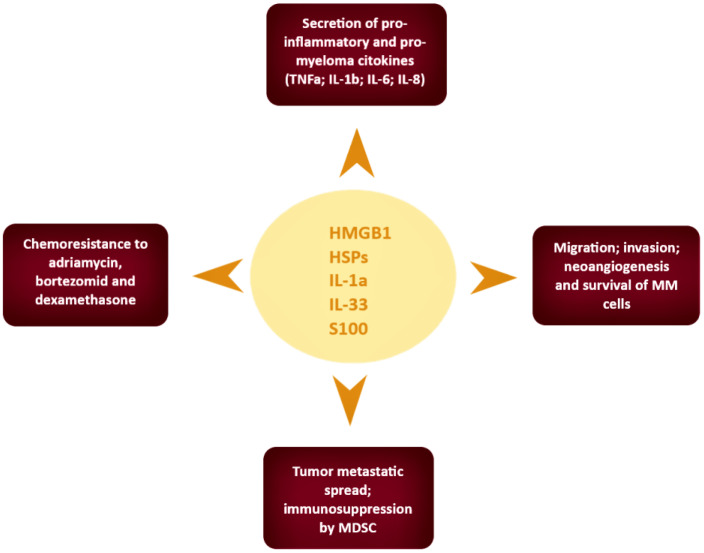
Role of alarmins in the pathogenesis of multiple myeloma.

It is now considered plausible that alarmins can activate similar intracellular signalling pathways to those of chemoattractants and chemokines by binding to different types of receptors. Through the signal induced by the binding with a specific receptor, the alarmins will be able to determine well definite biological effects [69]. For example, defensin (α, β) by binding to CCR2, CCR6, and TLR-4 carries out its antimicrobial action and leukocyte recruitment, and HMGB1—through binding to C-X-C motif chemokine receptor 4 (CXCR4), receptor of advanced glycation end products (RAGE), TLR-2, TLR-4, and TLR-9—regulates gene transcription and leukocyte recruitment [69]. The anti-cancer response determined by the innate/inflammatory immunity cells can induce anti-cancer immunity or cancer progression with the onset of metastases and concomitant immunosuppression. Alarmins are likely to play complex and multiple roles in cancer immunity by having different effects on leukocytes and the immune response. In Figure 1 we have summarized the main roles of alarmins in the pathogenesis of multiple myeloma. 

## 3. Search Strategy

We have selected scientific publications on the specific topic “alarmins, MGUS and MM” drawing from Pubmed. The keywords we used were alarmins, MGUS, MM, and immune system. The primary end point was to select research papers in which the role of one or more alarmins in MGUS and MM has been investigated. By carefully reading the individual articles, we identified the specific alarmins as well as their suggested role in the progression of MGUS in MM and in the onset of immunodeficiency secondary to MGUS and MM. Therefore, the secondary end point was to propose a possible network between the alarmins that can help in understanding one of the mechanisms capable of predicting the malignant evolution of the disease: how to prevent and possibly envisage future clinical research aimed at determining a possible target for monoclonal therapies. 

## 4. Results

We identified 16 research articles evaluating the most studied alarmins in patients with MGUS and MM reported in Table 1. We selected research articles evaluating HMGB1, HSPs, IL-1, S100, and IL-33. 

### 4.1. HMGB1-Induced Chemoresistance

Guo et al. [70] demonstrated that a high expression of HMGB1 from MM cell lines and primary MM can negatively impact the 3-year survival of patients with MM. Furthermore, HMGB1 blocks dexamethasone-induced MM cell apoptosis. All this confirms how the downregulation of HMGB1 favors cell apoptosis and increases the response to chemotherapy treatment. Furthermore, Ning et al. [71] investigated whether HMGB1 played a role in the chemoresistance to adriamycin, bortezomib, and dexamethasone of MM cells using the MMCL RPMI8266 cell line. However, adriamycin, bortezomib, and dexamethasone-resistant cells presented higher expression of HMGB1 and significantly decreased cell apoptosis compared with the parental RPMI8266. The restoration of the pharmacological sensitivity of the tumour cells is favored by the increase of NF-κB activity, confirming that HMGB1 regulates drug resistance in MM cells by regulating the NF-κB signalling pathway. Allegra et al. [72] analysed in 19 MM patients the sRAGE and AGE/sRAGE axis. Patients with MM had elevated sRAGE but decreased advanced glycation end products (AGE) values without any correlation with gender and age. Notably, sRAGE values were also related to the type of M protein produced by myeloma cells. Indeed, the concentration of sRAGE was higher in IgA Lambda MM, compared to IgA kappa, IgG kappa, and IgG Lambda. However, sRAGE is known as a factor capable of inducing the activation of osteoblasts having a protective role in bone disease progression. These findings confirmed that sRAGE might be a protective role in bone progression [73]. Huang et al. [74] investigated in MM cell lines (RPMI 8226, OPM2, and MM.1S) the effects of bortezomib, the first therapeutic proteasome inhibitor recommended for the first-line treatment in refractory, relapsed, and newly diagnosed MM. It has been postulated that the activation of the heat shock response may be one of the mechanisms by which MM does not respond or partially responds to bortezomib. However, bortezomib has been shown to upregulate HSP70 expression and, thus, the viability of MM cells. The authors proved that MM cell viability was decreased by inhibiting HSP70 with VER-155008. These findings confirmed that the synergistic action of bortezomib and VER-155008 could represent a therapeutic option favoring MM cell death. It has been hypothesised that the administration of intravenous immunoglobulin G may perform anti-tumour activity. Since there is a correlation between inflammation and tumours, it is likely that intravenous immunoglobulin G can slow down the growth of tumours due to their anti-inflammatory capacity. Confirming this, Jones et al. [75] demonstrated that intravenous immunoglobulin G has now been shown to contain IgG that block HSP70-1, thus inhibiting the growth of tumour cells both using MM and mantle cell lymphoma cells in vitro and with co-administering bortezomib in vivo.

### 4.2. IL-1β as a Progression Factor of MM

IL-1β now seems able to favor the progression of MM by acting through the activation of platelets. Takagy et al. [76] showed that platelets from MGUS, smoldering MM, and MM patients expressed significantly higher levels of P-selectin than platelets from healthy control subjects. Activated platelets release cytokines and growth factors including epidermal growth factor (EGF), platelet-derived growth factor (PDGF), growth-related oncogen protein-α (GRO), macrophage inflammatory proteins (MIP)-1 beta, macrophage migration inhibitory factor (MIF), and granulocyte colony-stimulating factor (G-CSF), which is able to induce cell proliferation also through the regulation of IL-1β signalling. In co-cultures of MM cells with activated platelets, elevated levels of IL-1β are observed, which arise from MM tumour cells and not from platelets themselves, which contain minimal amounts of IL-1β. Furthermore, increased IL-1 levels also go hand in hand with increased IL-22 levels in patients with MM, as demonstrated by Tsirakis et al. [77] in 51 active MM patients. Indeed, elevated levels of IL22, IL-1β, and beta-2 microglobulin were present in patients with active MM and correlated with disease stage and degree of infiltration, but not with levels of M protein. It is likely that IL-22 promotes the progression of MM and also plays a role in immune dysregulation. It should be remembered that IL-1, IL-6, transforming growth factor (TGF)-β, and IL-23 favor the differentiation of Th17 cells, which have a widely demonstrated role in the etiology of autoimmune diseases and allergic diseases [78,79].

### 4.3. Role of Proinflammatory Cytokines in MM Progression

However, Ben Hmid et al. [80] analysed the role of Th17 cells in 29 MM patients. The authors demonstrated that the gene expression of IL-17 and RAR Related Orphan Receptor C (RORC) correlated with each other, and it is accompanied by the increased expression of Th17 cells in the bone marrow of patients with MM as compared to healthy controls. These findings support the hypothesis of an active role of Th17 in the progression of MM. Musolino et al. [81] investigated IL-33 plasma levels in 13 MGUS and 44 MM patients. Plasma levels of IL-33 were significantly lower in MM patients and especially in an advanced stage of the disease. These findings confirm the importance of IL-33 in supporting the Th2 response against idiotype protein (Id) secreted by MM cells; thus, IL-33 plays a fundamental role in controlling the progression from MGUS to MM. It is conceivable that the decrease in IL-33 determines on the one hand the progression from MGUS to MM and on the other hand weakens the immune response not only towards tumour cells but also towards exogenous antigens, thus contributing to the genesis of secondary immunodeficiency. Osteolytic bone disease represents a complication able to favor the progression of MM. Zhu et al. [82] demonstrated that IL-33 inhibits the differentiation of osteoclast precursors into mature forms in cultured bone marrow cells with the receptor activator of NF-κB ligand (RANKL) plus macrophage-CSF (M-CSF). MGUS and MM induce a chronic inflammatory state in the host supported by the action of proinflammatory cytokines, which modifies the sialylation of the Ig fragment crystallisable (Fc) region of both M protein and PIg. Bosseboeuf et al. [83] analysed 148 MGUS and MM patients and demonstrated the higher concentration of IL-11, RANTES, hepatocyte growth factor (HGF), and stromal cell-derived factor 1 alpha (SDF-1-α) in patients with MM compared to those with MGUS. These findings could explain why monoclonal IgG in patients with MM are less sialylated than monoclonal IgG in subjects with MGUS. Furthermore, elevated levels of IL-6, IL-17, IL-33, TGF-β1, HGF, and TNF-α are associated with hyposialylated pc IgGs. 

### 4.4. S100 Protein as Responsible for Disease Progression

De Veirman et al. [84] analysed the potential role of extracellular S100A9 in MM using the 5T33MM immunocompetent mouse in which MM progression is characterised by an accumulation of MDSC in the BM and an increase in angiogenesis. The authors confirmed the ability of S100A9 to induce the secretion by myeloid-derived suppressor cell (MDSCs) of inflammatory and pro-myeloma cytokines including TNFα, IL-6, and IL-10. Furthermore, they also demonstrated that the combination of ABR-238901 and bortezomib decreases tumour progression and represents a valid therapeutic option. Notably, ABR-238901, a small molecule that inhibits the interaction of S100A9 with its receptors, decreases the secretion of IL-6 and IL-10 from MDSCs, inhibits the neoangiogenic process, but would not have shown satisfactory efficacy in reducing tumour progression. Bao et al. [85] investigated the S100A6 and Notch1 levels in 28 MM cases and 20 healthy controls. They confirmed that the levels of S100A6 and Notch1 were elevated in MM patients as compared to healthy controls and are associated with disease progression. In Table 1 we have reported the research articles analyzed.

## 5. Discussion

It is now clear how the relationship between MM cells and bone marrow stromal cells plays a role in determining the inflammatory state that would favor the progression of the disease and secondary immunodeficiency. Proinflammatory cytokines would play a critical role in promoting neoplastic progression. However, IL-1, IL-6, IL-12, IL-15, IL-16, IL-17, IL-18, IL-22, IL-23, TNF-α, and IFN-γ, which are assayed in serum or bone marrow of patients with MM have a proinflammatory action. In contrast, IL-1Rα, IL-4, IL-10, IL-11, TGF-β1, HSPs, and lipoxin A4 have an anti-inflammatory action. Surely, the listed cytokines allow the crosstalk between the bone marrow stroma and the plasma cells inducing resistance to cell death stimuli and downregulating differentiation markers, thus favoring malignant plasma cell phenotype [86,87,88]. It should be remembered that the progression and metastatic spread of MM occurs as in all tumours when immunosurveillance is won through, first of all, immunoediting [89].

### 5.1. Role of the Immune System in MM Progression

The presence of T cells specific for the idiotype of the monoclonal paraprotein is described in patients with MGUS and MM, although there is still no definitive evidence on their actual role [90,91]. IL-1 plays a pivotal role in the progression from MGUS to MM as well as having a proinflammatory role. It is now well established that the plasma cells of patients with MM exhibit high concentrations of IL-1β. On the contrary, IL-1β is not detectable in the plasma cells of patients with MGUS. Therefore, the productive switch of IL-1β would represent a signal of malignant progression of the disease [92,93]. Confirming its probable role in the progression from MGUS to MM, IL-1β would seem to enhance the ability of plasma cells to cause lytic bone lesions, to induce the expression of adhesion molecules such as ICAM, CD44, CD54, CD56, CD44, and VLA-4 on plasma cells by increasing their capacity for transmigration and metastatic widespread [94,95,96,97,98]. It is hypothesised that a viral infection may represent the initial stimulus capable of inducing the upregulation of IL-1β. Among the viruses involved, there would be Kaposi’s sarcoma-associated herpesvirus, Epstein–Barr virus, human immunodeficiency virus-1, and respiratory syncytial virus [99,100]. An interesting finding that could give a further potential explanation of the cytokine network that favors disease progression would be related to the increased expression of IL-2R on MM cells and to a lesser extent on MGUS cells, suggesting that progression from MGUS to MM is also related to the alteration of the IL-2/IL-2R system [101]. 

### 5.2. Neoangiogenesis in MM

The elevated expression of HMGB1 has an undoubted role in the transformation of MGUS into MM and in the progression of MM itself, blocking cell apoptosis and inducing drug resistance by regulating the NF-κB signalling pathway. Notably, HMGB1 induces neoangiogenesis in patients with solid tumours and other hematological neoplasms. Meyer et al. [102] demonstrated in 18 non-Hodgkin lymphomas and two lymphoma cell lines that HMGB1 probably released from necrotic cells promotes tumour neoangiogenesis in a paracrine way. Zhan et al. [103] confirmed that autophagy induced the release of HMGB1 by gastric cancer cells into the extracellular space after exposure to vincristine, protecting gastric cancer cells from apoptosis through the upregulation of Mcl-1. Shrivastava et al. [104] demonstrated that elevated levels of HMGB1 reduced the response to radiotherapy in eight human urothelial carcinoma cell lines. Zhang et al. [105] confirmed HMGB1 expression in non-small cell lung cancer cell lines and that exposure to adriamycin, cisplatin, and methotrexate further increased HMGB1 levels. However, Chen et al. [106] confirmed the ability of HMGB1 to protect chronic myeloid leukemia cells from apoptosis, as proven in other studies [107,108,109,110]. Wu et al. [111] analysed the impact of HMGB1 expression on clinical progression in 166 patients with nasopharyngeal carcinoma. The study showed that the high expression of HMGB1 increased the risk of metastatic disease with poor prognosis. Süren et al. [112] confirmed the negative impact of HMGB1 in 110 patients with colorectal carcinoma. Several studies have shown that HMGB1 blockade restores apoptosis. It is plausible that HMGB1 promotes neoangiogenesis through the expression of vascular endothelial growth factor (VEGF) as occurs in chronic immune-mediated diseases and allergies such as allergic rhinitis [113,114]. Ostecytes play a key role in the progression of MM by promoting neoangiogenesis through the expression of molecules, including VEGF [115]. It is now shown that VEGF by stimulating its type 2 receptor (VEGFR2) induces neoangiogenesis, favoring the progression of MM [116]. Single nucleotide polymorphisms (SNPs) of VEGF and VEGFR2 genes have been demonstrated to play a key role in MM progression. For example, the presence of the VEGFR2-604TT genotype is associated with stage II or III tumours but not with stage I tumours [116]. Furthermore, endothelial dysfunction that accompanies neoangiogenesis in turn favors the passage of immune cells into the tumour environment through the expression of direct chemokines and cell adhesion molecules on the surface of endothelial cells as also occurs in chronic immune-mediated diseases [117,118].

### 5.3. Drug Resistance in MM

Crosstalk between adaptive immune cells and the endothelium is critical to tumour immune surveillance and the success of immune-based therapies that use immune cells to kill tumour cells. There are several factors that can reduce the pharmacological response of MM, resulting in disease progression. Notably, MicroRNAs (miRNAs) are an abundant group of endogenous non-coding RNAs (about 22 nt) binding to target mRNAs mainly at their 3′-untranslated (UTR) that displayed a key role in lung cancer [119]. However, miR-218 can inhibit the metastatic spread of tumours including non-small cell lung cancer and pancreatic cancer by blocking the expression of HMGB1 [120,121]. However, miRNAs appear to play a role in resistance to chemotherapy drugs. Ran et al. [122] demonstrated that the downregulation of miRNAs is associated with resistance to paclitaxel in endometrial cancer cells. Notably, miR-218 binds directly to the 3’-UTR of the HMGB1 gene, the upregulation of which induces resistance to paclitaxel in endometrial cancer cells by promoting autophagy. MiR-218 overexpression would inhibit HMGB1-induced autophagy by restoring tumour cell response to paclitaxel.

### 5.4. MGUS to MM: Role of Immunoglobulin Chains

The roles that the quantity and type of M protein and the ratio of free immunoglobulin chains have in the evolution of the disease from MGUS to MM is now widely accredited. It is also evident that the suppression of the production of PIg is not only a progression factor but also has a negative impact on the clinical evolution favoring “immune paresis” and consequently increasing the risk of opportunistic infections [33,68]. Rapid progression from MGUS to MM is favored by the decreased synthesis of IgM in subjects with IgG or IgA gammopathy, and of IgA in individuals with IgG or IgM gammopathy [123,124,125]. The network that influences immune paresis is certainly complex, and it is not yet completely clear whether it all starts from the dysregulation of cytokines and whether this dysregulation is due to defects in immune surveillance, the proliferation of clonal plasma cells, or opportunistic infections [126]. Moreover, the immune paresis reaches its peak with old age but decreases among the very elderly [127]. HMGB1 facilitates autophagy by disrupting the interaction between Beclin 1 and its downregulator Bcl2 via competitively binding to Beclin 1 [128]. Wang et al. confirmed that increased HMGB1 expression in the neuroblastoma NB SH-SY5Y cell line induced resistance to treatment with doxorubicin, etoposide, and cisplatin. Sensitivity to treatment was restored after HMGB1 was knocked out by RNA interference [112]. HMGB1 has been shown to induce elevated levels of LC3-II (a protein light chain associated with microtubules that plays a role in autophagosome formation) and the selective degradation of p62 (a ubiquitin-related protein) in autophagy. In this way, the green fluorescent protein-light chain 3 favors the generation of autophagosomes [112].

### 5.5. HMGB1/RAGE Axis as Crosstalking between Immune Cells and Bone Tissue

**Figure 2:**
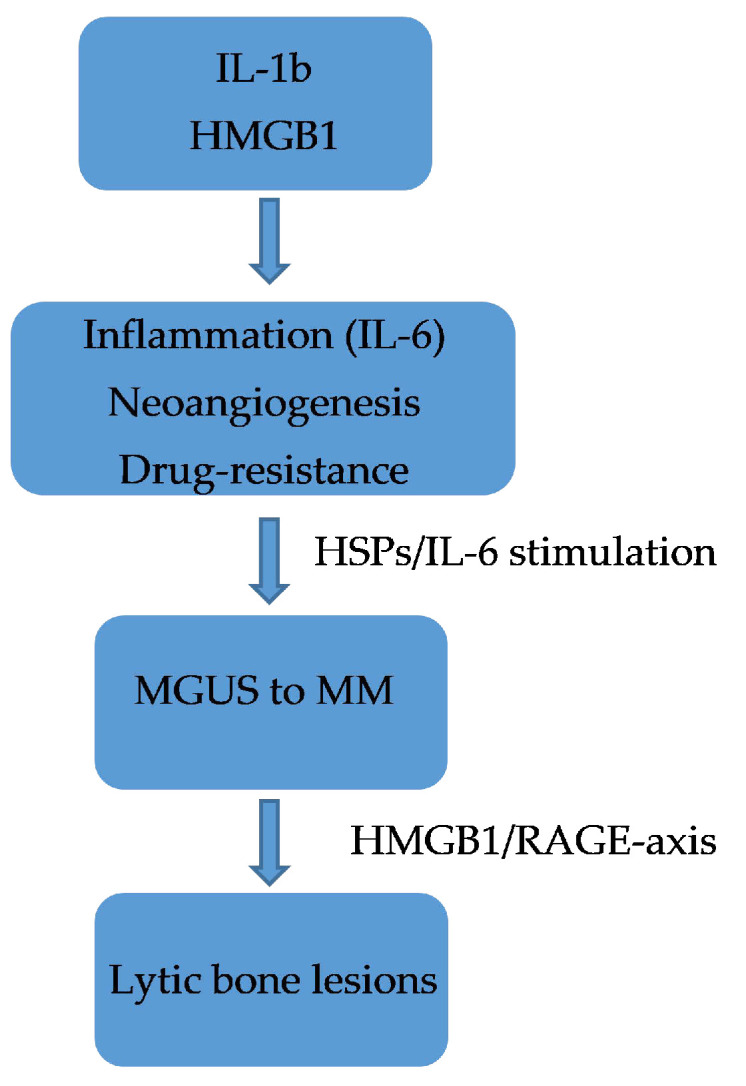
Crosstalking between immune cells and bone tissue in multiple myeloma.

In patients with pancreatic cancer, the mechanism of action of HMGB1 is not yet fully understood, although it seems to have a dual action. It is likely to inhibit tumourigenesis by stabilising the genome through its direct binding to p53 at the intracellular level and by binding to certain receptors including RAGE, TLR-2, TLR-4, and TLR-9 as well as by interacting with cytokines and growth factors at the extracellular level [113]. In patients with MM, a plant-based chemical lycorine has been shown to be able to decrease HMGB1 and, therefore, LC3 and Beclin-1, thereby blocking autophagy. In particular, lycorin dissociates Bcl2 from Beclin-1 by acting through the ubiquitin–proteasome pathway [128]. Lycorin restores the response of MM patients to bortezomib treatment by reducing HMGB1 expression. These data confirm that lycorine has a dual activity: both antitumourigenic and promoting sensitisation to chemotherapy in MM [129]. There is no doubt that the not too distant future approach will be to design and synthesise monoclonal antibodies directed against specific molecules including HMGB1 to make therapies targeted and hopefully with fewer adverse effects [130]. Confirming this, Usman et al. [131] have reported some clinical trials targeting HMGB1. RAGE is among the receptors to which HMGB1 binds, and it is a member of the immunoglobulin superfamily and is a transmembrane receptor that binds to AGE. HMGB1 activates RAGE or activates the peroxisome via the proliferator-activated receptor gamma (PPAR-γ) with consequent inhibition of the HMGB1-RAGE axis, and this mechanism could also represent a future therapeutic strategy beneficial against tumours [73,74]. Furthermore, CXCR4 expression is ubiquitous in different hematopoietic cells [132], and its role in the evolution of MM has already been demonstrated [133]. It is now well established that elevated levels of CXCR4, integrins (i.e., CD11a/CD11c/CD29/CD49d/CD49e), and adhesion molecules (i.e., CD44/CD54) in patients with MM induce resistance to chemotherapy [134,135]. Furthermore, the HMGB1/RAGE axis has now been shown to be able to favor bone loss. HMGB1 is expressed in primary osteoblasts and osteoclasts both expressing RAGE. Furthermore, the RANKL/osteoprotegerin (OPG)/receptor activator of NF-κB (RANK) signalling axis plays a critical role in DCs function as well as bone remodeling. RANKL is present in DCs that release HMGB1 and represents a mode of crosstalking between immune cells and bone tissue. These data would confirm the complexity of the mechanisms and the role of the bone marrow microenvironment in the progression of MM [136,137]. RAGE is encoded in the Class III region of the major histocompatibility complex and it is the multiligand receptor of the immunoglobulin superfamily. RAGE stimulated by AGEs has been shown to trigger pro-tumourigenic pathways by promoting the dissemination of melanoma and pancreatic cancer cells [137] and probably has a similar action also in colorectal, oesophageal, and oral squamous cell carcinoma, since in these tumours, it is upregulated and, therefore, acts as an oncoprotein [113,114]. The fact that levels of sRAGE are higher in the IgA Lambda MM, which has a poorer prognosis, is explained as a reactive mechanism to the tumour, which is fully confirmed by the ability of the same sRAGE to bind other ligands such as proinflammatory molecules. There is no doubt that HSPs also play a role in the activation of proinflammatory cytokines secreted by monocytes/macrophages and favor the activation of immature DCs [65,118]. IL6 increases the expression of HSP90 in patients with MM, thus favoring the survival of myelomatous plasma cells. It is now shown that the increase in HSP90 levels is induced by STAT3 and CCAAT/enhancer-binding protein β (C⁄EBPβ), which bind to and activate the HSP90β promoter [76]. In Figure 2 we have summarized the crosstalking between immune cells and bone tissue in multiple myeloma. 

## 6. Mutational Status, Inflammation, and MM Progression

Similar to all tumours, MM is a condition caused by genetic alterations capable of both inducing the disease and influencing its prognosis. It remains to be assessed whether even in MM the inflammatory condition may be able to determine a particular mutational state capable of favoring the disease or whether genetic mutations of MM can affect an inflammatory condition. For instance, a characteristic of myelodysplastic syndromes (MDSs) is stimulation of the NLRP3 inflammasome, which provokes clonal proliferation and pyroptotic cell death. MDS hematopoietic stem cells overexpress inflammasome proteins that direct the generation of interleukin-1β, IL-18, and pyroptotic cell death. Mechanistically, pyroptosis is triggered by the alarmin S100A9 that is found in excess in MDS HSPCs and BM plasma. Further, similar to somatic gene mutations, S100A9-induced signalling activates NADPH oxidase (NOX), increasing levels of reactive oxygen species (ROS) that initiate cation influx, cell swelling, and β-catenin activation. Notably, the knockdown of NLRP3 or caspase-1, neutralisation of S100A9, and pharmacologic inhibition of NLRP3 or NOX suppress pyroptosis, ROS production, and nuclear β-catenin in MDSs and are sufficient to restore effective hematopoiesis. Thus, alarmins and founder gene mutations in MDSs license a common redox-sensitive inflammasome circuit, which proposes new approaches for therapeutic intervention [138].

Similarly, transcriptomic studies of hematopoietic stem cells from patients affected by myeloproliferative neoplasms (MPNs) with driver mutations have established the upregulation of inflammation-related genes able to cause the development of an inflammatory state. The possibility of operating on the inflammatory state as a therapeutic approach in MPNs appears promising, in which an intervention acting on the pathways that control the synthesis of cytokines and oxidative stress could be successful in decreasing the possibility of leukemic progression [139].

As far as the relationship between immune response, inflammation, and mutational status, MM subjects with the same genetic alterations are inclined to have both plasma cells and immune cells clustered together [140]. 

Numerous genomic analyses have led to a better understanding of the molecular pathogenesis of myeloma. These reports have verified recurrent mutations in KRAS, NRAS, and TP53 as well as a significant percentage of previously unknown mutations influencing RNA processing and protein homeostasis [141]. 

In a study, Liu et al. validated AP-1 complex differential expression (JUN and FOS) in plasma cell subpopulations using mass cytometry (CyTOF)-based protein assays. Integrated analysis of single-cell RNA and CyTOF data revealed activator-protein-1 (AP-1) downstream targets (IL6 and IL1B) potentially leading to inflammation regulation. Thus, the close link between gene alterations, immune response, and inflammation in MM is further highlighted [142].

Furthermore, as far the role of alarmins in MM progression and the possibility of their use as biomarkers to predict MGUS evolution or disease relapse in patients, a recent report indicated that a significant augmented risk for being diagnosed with MGUS was linked to inflammatory disorders, while inflammatory signalling has a central role in MSC transformation by causing a pro-tumour phenotype correlated with a permissive BM milieu permitting immune escape and MM growth [143].

Finally, Botta et al. investigated the relevance of inflammatory genes in predicting disease evolution and patient survival. They performed a bioinformatics study on a gene expression profiling dataset of MGUS, smoldering MM, and symptomatic MM, and they recognised inflammatory and cytokine/chemokine pathways as the most gradually modified during disease evolution. They identified an eight-gene signature (IL8, IL10, IL17A, CCL3, CCL5, VEGFA, EBI3, and NOS2), distinguishing each condition (MGUS/smoldering/symptomatic MM) with 84% accuracy. Moreover, six genes (IFNG, IL2, LTA, CCL2, VEGFA, CCL3) were reported independently correlated with patient survival. Subjects whose MM cells presented high concentrations of Th1 cytokines (IFNG/LTA/IL2/CCL2) experienced the longest survival. On these genes, they developed a prognostic risk score. In this study, they furnished a proof that inflammation has an essential effect in MM patient progression and survival. The inflammatory gene prognostic signature evidently suggests new prospects for personalised anti-MM therapy [144].

Similar studies performed on alarmins will definitively clarify the possibility of using these molecules as biomarkers and prognostic factors in monoclonal gammopathies.

## 7. Conclusions and Future Directions

Is it possible to think as a future prospect the possibility of blocking HSPs to slow the progression from MGUS to MM or to slow down the metastatic progression of MM? The answer is not yet satisfactory and certain, but some attempts open to positive perception. Unfortunately, a considerable portion of patients with MM still relapse and, therefore, require new therapies, moreover, reducing the duration of response to each therapeutic regimen over time. In particular, relapsed and refractory MM (RRMM) can make use of new molecules capable of inhibiting histone deacetylase (HDACis), including panobinostat, which influences transcriptional activation and other nuclear events by increasing histone acetylation [145].

Panobinostat combined with bortezomib and dexamethasone blocks the chaperone function of HSP90, resulting in slowdown in MM cell proliferation [145,146,147]. Notably, molecules that inhibit HDACis block angiogenesis and osteoclastogenesis, reducing the secretion of VEGF and hypoxia-inducible factor-1 (HIF1)-α as well as inducing the degradation of all three VEGF receptors [145,146,147,148]. S100A9 also plays a role in the bone marrow microenvironment to induce angiogenesis and promote the progression of MM. De Veirman et al. [149] reported that that the molecule ABR-238901 blocks the activity and interactions of S100A9 by inhibiting the expression of IL-6 and IL-10 by MDSCs. Finally, IL-33, a member of the IL-1 family constitutively expressed by endothelial and epithelial cells, seems to guide Th2 response in several organs. However, the decreased response to exogenous antigens is accompanied by the presence of hyposialylated PIg in MGUS and MM patients, presenting high levels of HGF, IL-6, TNF-α, TGF-β1, IL-17, and IL-33 [32,66]. It is now generally accepted that neoangiogenesis is induced by stimuli that arise in the medullary microenvironment, including cells of the immune system, APCs, cytokines, and alarmins. Finally, modulation of the host immune system and the inhibition of neoangiogenesis may represent the therapeutic target for the treatment of MM capable of promoting better survival and reducing the risk of RRMM.

In conclusion, the tumour microenvironment is a composite system of tumour and non-tumour cells, as well as of signalling molecules that can modify anti-tumour immune responses, supporting MM proliferation and maintenance. With growing evidence underlining the effect of alarmins in MM progression, these elements are an interesting target for MM treatment. Modulators of alarmins have targeted several characteristics of their biology, from the inhibition of their production and their interaction with other proteins to blockading their activity, and there are many new directions through which research could turn. For example, Zhao et al. evaluated the significance of neutrophils and the neutrophil-derived DAMP protein, MRP14, in antibody delivery [150]. Splenic neutrophils and MRP14 that are present in the splenic peri-marginal zone (MZ) region have a tight correlation with MZ B cells and stimulate their maturation into plasma cells. Employing neutrophil-depleting animals and an MRP14-stopping substance, they demonstrated that the presence of neutrophil and MRP14 is essential for class switch, plasma cell maintenance, and antibody generation. Moreover, they reported that MRP14 could also be delivered by neutrophils in the BM and support the maintenance of BM plasma cells. MRP14 binding could increase the effect of the BAFF signal and defend primary multiple myeloma cells from doxorubicin-induced apoptosis. Therefore, an inhibition of this specific signalling could be useful in the treatment of MM.

Another fascinating field of study could be the analysis of abnormal circulating extracellular vesicles (EVs) stemming from altered endosomal–lysosomal system. The relevance of EVS has also been reported in MM, and it has been indicated as a promising route for therapeutic purposes [151]. However, whether there is a common link present between DAMPs and EVs production is yet to be elucidated. A greater comprehension of the intricate and variable intracellular and extracellular trafficking of DAMPs and EVs, comprising those of mitochondrial origin, is essential to disclose relevant pathogenic pathways and new targets for novel treatments [151,152].

## Figures and Tables

**Table 1:** Research articles analyzed and their main results.

Author	Year	Type of Study	Objective	Outcome
Xing Gu, Donghua He, Enfan Zhang et al.	2018	Research paper	Evaluate the effect of HMGB1 and the mechanism involved in multiple myeloma drug resistance	HMGB1 may serve as a target for MM treatment
Jing Ning, Rui Yang, Hainan Wang, Lijuan Cui	2021	Research paper	Explore the exact molecular mechanism underlying HMGB1-mediated drug resistance in multiple myeloma	HMGB1 regulates drug resistance in MM cells by regulating NF-κB signalling pathway
Alessandro Allegra, Caterina Musolino, Elisabetta Pace et al.	2019	Research paper	Evaluate the advanced glycation end products/soluble receptor of advanced glycation end products (AGE/sRAGE) axis in patients with multiple myeloma (MM)	Serum concentrations of AGE and sRAGE could therefore become potential therapeutic targets in multiple myeloma
E Galliera, M G Marazzi, E Vianello et al.	2016	Research paper	To investigate the diagnostic potential of sRAGE to improve the detection and monitoring of bone metastasis	sRAGE might play a protective role in bone metastasis progression, and it may have diagnostic significance for detecting and monitoring osteolytic metastases
Lingjuan Huang, Yanmeng Wang, Ju Bai et al.	2020	Research paper	To investigate whether targeting HSP70 using a specific inhibitor VER-155008 combined with bortezomib could overcome the acquired resistance in multiple myeloma	Find of a strong synergistic interaction between VER-155008 and bortezomib may support for combination therapy in multiple myeloma patients
Richard J Jones, Ram K Singh, Fazal Shirazi et al.	2020	Research paper	To support the possibility that anti-HSP70-1 IgG contained in IVIgG can inhibit myeloma and MCL growth by interfering with a novel mechanism involving uptake of exogenous HSP70-1 which then induces its own promoter	IVIgG has a potential road map to identify new therapies that could be generated as monoclonal antibodies
Satoshi Takagi, Shokichi Tsukamoto, Jihye Park et al.	2018	Research paper	To investigate the association of platelet activation status with clinical stages in multiple myeloma (MM) patients and explored the role of platelets in MM progression	Platelets from MM patients were highly activated with disease progression. IL-1β is critical to platelet-mediated MM progression and might be a potential target for MM treatment
George Tsirakis, Constantina A Pappa, Anna Kolovouv et al.	2015	Research paper	To estimate serum levels of IL-22 in MM patients, both in activity and remission, in order to apprehend its possible participation in MM biology	Elevated levels of IL-22 in active MM patients, in parallel with disease activity, and in positive correlation with IL-1beta, may represent the inflammatory element of the disease
Murdaca G, Colombo BM, Puppo F	2010	review	Focus on recent information regarding IL-17 and its relevance in autoimmune and chronic inflammatory diseases	IL-17 plays a key role in various steps of RA, SLE, and other autoimmune and chronic inflammatory diseases development, and it is associated not only with T cell-mediated tissue injury but also with the production of pathogenic autoantibodies
G Ciprandi, M De Amici, G Murdaca, D Fenoglio, F Ricciardolo, G Marseglia, M Tosca	2009	Research paper	To investigate a possible relationship between serum IL-17 levels and clinical parameters in patients with allergic rhinitis studied during the pollen season	Serum IL-17 levels were significantly related to clinical symptoms, drug use, and peripheral eosinophil counts
Ahlem Ben Hmid, Oumayma Selmi, Raja Rekik et al.	2020	Research paper	Try to understand the role of Th17 lymphocytes in multiple myeloma	The involvement of Th17 cells in the pathophysiology of MM. Such data further support the use of anti-IL-17 antibodies as a therapeutic approach in MM
Caterina Musolino, Alessandro Allegra, Mirella Profita et al.	2013	Research paper	To demonstrate decreased concentrations of IL-33 in patients with MM	Reduced IL-33 levels might prevent an adequate Th2 response towards MM idiotype proteins, and compromise the anti-tumour surveillance
Xiaoqing Zhu, Yinghua Zhao, Yuxue Jiang et al	2017	Research paper	Curdlan potently inhibited RANKL-induced osteoclast differentiation and the resultant bone resorption	IL-33 receptor, partially abrogated curdlan-induced inhibition of NFATc1 expression and osteoclast differentiation
Adrien Bosseboeuf, Sophie Allain-Maillet, Nicolas Mennesson et al	2017	Research paper	Characterised the sialylation of purified mc and pc IgGs from 148 MGUS and MM patients, in comparison to pc IgGs from healthy volunteers	Hyposialylation of mc IgGs contribute to the pathogenesis of MGUS and MM
Kim De Veirman, NathanDe Beule, Ken Maes et al	2017	Research paper	To evaluated the role of extracellular S100A9 and the therapeutic relevance of S100A9 inhibition in multiple myeloma (MM), using the immunocompetent murine 5T33MM model	Extracellular S100A9 promotes MM and that inhibition of S100A9 may have therapeutic benefit
H Y Bao, Y Wang, J N Wang, M Song, Q Q Meng, X Han	2017	Research paper	To investigate the expression levels of S100A6, Notch1 in multiple myeloma (MM) patients and its clinical significance	S100A6 and Notch1 were closely associated with MM progress and intramedullary metastasis

## Data Availability

Not applicable.
